# μ- PBWT: a lightweight r-indexing of the PBWT for storing and querying UK Biobank data

**DOI:** 10.1093/bioinformatics/btad552

**Published:** 2023-09-09

**Authors:** Davide Cozzi, Massimiliano Rossi, Simone Rubinacci, Travis Gagie, Dominik Köppl, Christina Boucher, Paola Bonizzoni

**Affiliations:** Department of Informatics, Systems and Communication, University of Milano-Bicocca, Milan 20126, Italy; Department of Computer & Information Science & Engineering, Herbert-Wertheim College of Engineering, University of Florida, Gainesville, Florida 32611, United States; Department of Computational Biology, University of Lausanne, Lausanne 1015, Switzerland; Faculty of Computer Science, Dalhousie University, Halifax B3H 4R2, Canada; M&D Data Science Center, Tokyo Medical and Dental University, Tokyo 113-8510, Japan; Department of Computer Science, University of Muenster, Muenster 48149, Germany; Department of Computer & Information Science & Engineering, Herbert-Wertheim College of Engineering, University of Florida, Gainesville, Florida 32611, United States; Department of Informatics, Systems and Communication, University of Milano-Bicocca, Milan 20126, Italy

## Abstract

**Motivation:**

The Positional Burrows–Wheeler Transform (PBWT) is a data structure that indexes haplotype sequences in a manner that enables finding maximal haplotype matches in *h* sequences containing *w* variation sites in O(hw) time. This represents a significant improvement over classical quadratic-time approaches. However, the original PBWT data structure does not allow for queries over Biobank panels that consist of several millions of haplotypes, if an index of the haplotypes must be kept entirely in memory.

**Results:**

In this article, we leverage the notion of r-index proposed for the BWT to present a memory-efficient method for constructing and storing the run-length encoded PBWT, and computing set maximal matches (SMEMs) queries in haplotype sequences. We implement our method, which we refer to as μ-PBWT, and evaluate it on datasets of 1000 Genome Project and UK Biobank data. Our experiments demonstrate that the μ-PBWT reduces the memory usage up to a factor of 20% compared to the best current PBWT-based indexing. In particular, μ-PBWT produces an index that stores high-coverage whole genome sequencing data of chromosome 20 in about a third of the space of its BCF file. μ-PBWT is an adaptation of techniques for the run-length compressed BWT for the PBWT (RLPBWT) and it is based on keeping in memory only a succinct representation of the RLPBWT that still allows the efficient computation of set maximal matches (SMEMs) over the original panel.

**Availability and implementation:**

Our implementation is open source and available at https://github.com/dlcgold/muPBWT. The binary is available at https://bioconda.github.io/recipes/mupbwt/README.html.

## 1 Introduction

Improved haplotype phasing in large cohorts is facilitating the comprehensive collection and study of variations at the chromosome level for genome evolution and clinical applications. This has been demonstrated by the haplotype-resolved whole-genome sequence data collected from hundreds of thousands of individuals for projects such as the UK Biobank (Halldorsson *et al.* 2022) and TOPMed projects (Taliun *et al.* 2021). In the field of phased genomics, the Positional Burrows–Wheeler Transform (PBWT) is a data structure that stores a set of *h* sequences containing *w* variation sites in a h×w binary matrix M[1..h][1..w], where the rows of M are sorted in co-lexicographic order (i.e. sorted order from right to left). It was initially proposed by Durbin (2014) as a means to find maximal haplotype matches in O(hw)-time, which abstractly can be seen as finding set maximal exact matches (SMEMs) in M, i.e. the longest common matching substrings between an external sequence *P* and any other sequence of the same length in M.

Despite the advantages of the PBWT for analyzing pangenomic haplotype data, it is relatively unknown in the data structures community. Efficient construction and representation of the PBWT on large datasets is in a relatively nascent stage by comparison to the BWT. Nonetheless, the PBWT has been applied and extended in numerous ways. It has been used for genotype imputation (Rubinacci *et al.* 2020), and to create a genotype database search method that is privacy-preserving (PBWT-sec) (Shimizu *et al.* 2016). Novak *et al.* (2017) and Sirén *et al.* (2020) used the PBWT to encode a graph for haplotype matching (g-PBWT) and graph pangenome indexing (Baaijens *et al.* 2022). Sanaullah *et al.* (2021) replaced all arrays with linked lists to define a dynamic version of the PBWT (d-PBWT). The original PBWT has been used to compute all-pairs Hamming distances (Mäkinen and Norri 2019) and for finding all maximal perfect haplotype blocks in linear time (Alanko *et al.* 2020). Notwithstanding the prior developments on the PBWT, analysis of large haplotype data—such as the UK Biobank data—using the PBWT remains a challenge. In November 2022, Jared Simpson tweeted: *What is the largest publicly available haplotype reference panel 1000 genomes? I’m looking for a pre-built PBWT index but don’t want to go through dbGAP to get the HRC panel*. Unfortunately, there are no sufficient solutions to this question.

In this article, we aim to address this by providing a means to efficiently build a compact PBWT in a manner that it retains the analysis goals of Durbin (2014). Our solution is to run-length encode the PBWT by exploiting in the PBWT framework, the data structure proposed in Rossi *et al.* (2022) for the BWT which allows efficiently finding SMEMs. Run-length encoding is a concept that was originally created to increase the space efficiency of the BWT, and is defined as storing the BWT in space that is proportional to the number of *runs* in the BWT. The number of runs is routinely denoted as *r*, where *r* is usually significantly smaller than *n* on repetitive input. Mäkinen and Navarro (2004) noticed that the BWT can be stored in O(r) space while still efficiently supporting some standard queries (i.e. count). Then, Gagie *et al.* (2020) showed how to augment the run-length compressed BWT with suffix array samples at the beginning or end of each run, such that it can support Φ queries which, given a suffix array entry, return the preceding suffix array entry; they called their data structure the r-index. The r-index is able to efficiently support the same queries as the FM-index and requires O(r) space. Rossi *et al.* (2022) then demonstrate how to make additions to the r-index to support finding SMEMs. Here, we propose and implement a data structure for storing the PBWT that is able to reduce the space of Durbin’s PBWT indexing (which is O(n)-space) in a manner that efficiently supports both finding and locating all SMEMs in the haplotype matrix.

In this article, we show how an r-indexing can be fully explored and extended to the PBWT of a panel of haplotype data that allows for both compact storage and efficient support of SMEM queries.

We implement our solution, which we refer to as μ-PBWT, and compare μ-PBWT to Durbin’s PBWT (Durbin 2014) and the best current PBWT index that allows matching queries, Syllable-PBWT (Wang *et al.* 2023) on 1000 Genome Project (The 1000 Genomes Project Consortium 2015). We demonstrate that μ-PBWT produces memory requiring from 1.1 to 25 times less space than those produced by Syllabe-PBWT. Compared to Durbin’s Algorithm 5, μ-PBWT uses up to 80 times less space at the cost of up to 2× increase in construction and query time for 100 queries, having a worst 6× increase in time with 1000 queries.

The experiments show that the best performance of μ-PBWT is achieved on whole genome sequencing (WGS) data of the UK Biobank. Indeed, μ-PBWT produces an r-index of 11.06 GB for chromosome 20 data, originally stored in a 29.6 GB BCF file; thus, in a third of the space of the BCF file format, which is already a significant compression of the input VCF file, we are able to store the data and keep all the data structures needed for computing SMEMs queries.

All these results show the scalability of μ-PBWT to current Biobank data when considering whole genome data, thus demonstrating the effectiveness of our tool in building and making easily available an index for downstream analysis of large genomic datasets.

## 2 Preliminaries

### 2.1 Positional Burrows–Wheeler Transform

We define a sequence *S* over a finite, ordered alphabet $\Sigma =\{c_{1},\ldots ,c_{\sigma }\}$ of σ characters to be the concatenation of *n* characters S=S[1..n]. We denote the empty sequence as ε. We denote the *i*th prefix of *S* as *S*[1.*i*], the *i*th suffix as *S*[*i*.*n*], and the sequence spanning position *i* through *j* as *S*[*i*.*j*], with S[i..j]=ε if i>j.

The PBWT has been introduced by Durbin as a data structure for handling a matrix M, representing a set $S=\{S_{1},\ldots ,S_{h}\}$ of *h* sequences of length *w* and over a binary alphabet, simply called haplotypes, by updating two arrays for each column *j*: the *prefix array* $PA_{j}$ and the *divergence array* $DA_{j}$. In this context, it is assumed that each variation site is bi-allelic, meaning that there exist only two observed alleles at a locus in the genome and no insertions or deletions. Although this binary encoding of genetic information appears to remove significant information, it is common practice in the analysis of variations of diploid species, where variations are filtered to only contain bi-allelic sites (Pinto *et al.* 2013, Zhu *et al.* 2018).

1.
$PA_{j}$ is the ordering of {1,…,h} induced by the co-lexicograph ordering of prefixes of *S* up to column j−1, i.e. formally $PA_{j}[i]=k$, if $S_{k}[1..j-1]$ is the *i*th element in co-lexicographically ordered list of prefixes $S_{1}[1..j-1],\ldots ,S_{h}[1..j-1]$.
2.
$DA_{j}[i]$ stores the length of the longest common suffix between the sequences of index $PA_{j}[i]$ and $PA_{j}[i-1]$ up to the (j−1)th column.

The PBWT of M is another matrix PBWT[1..h][1..w] that has the first column identical to the one of M while the *j*th column of M with j>1 is obtained by stably sorting the rows of M[1..h][1..j−1] in co-lexicographic order. To ease the notation we denote the PBWT of M simply as PBWT. Assuming to denote the *j*th column of a matrix M by $\mathrm{col}{\left(M\right)}_{j}$, formally $\mathrm{col}{\left(\mathrm{PBWT}\right)}_{1}=\mathrm{col}{\left(M\right)}_{1}$ and $\mathrm{col}{\left(\mathrm{PBWT}\right)}_{j}[i]=\mathrm{col}{\left(M\right)}_{j}[PA_{j}[i]]$ for all i=1..h and j=2..w.

The main idea is that the prefix-array PA defined above stores in each column *j* the permutation of the rows induced by a co-lexicographic ordering of the previous columns up to column j−1 while the divergence array DA stores in column *j* and position *i* the length of a longest common suffix between row *i* and the previous one in the permutation induced by the prefix array in column *j*. Together these two arrays allow to efficiently compute matching queries over haplotype sequences. Observe that we frequently use n=h⋅w to bound the space- and time- complexity.

If we consider the PBWT shown in Supplementary Fig. S1b and Column 5, then DA[5][7]=3 because the co-lexicographically 6th and 7th row prefixes (corresponding to PA[5][6]=18 and PA[5][7]=16 rows in the input matrix) up to Column 4 are 0100 and 1100 and their longest common suffix 100 has length 3.

### 2.2 Run-length encoded PBWT

We denote the run-length encoded PBWT matrix as RLPBWT. This extension is made by observing that the concept of *run* can be defined for the PBWT, i.e. the number of runs in the PBWT as the number of binary substrings containing occurrences of the same symbol which are maximal in length. Given $r_{j}$ as the number of runs in RLPBWT Column *j*, we denote *r* as $\sum_{1\leq j\leq w}r_{j}$. In the following, we will use the term PBWT without a specific distinction with the RLPBWT, as the RLPBWT distinguishes for the components it uses.

### 2.3 Set-maximal exact matches

One of the fundamental tasks of the PBWT is one-vs-all set-maximal exact matches (SMEMs) finding: the main idea is finding the longest common matching substrings between an external sequence *P* and any other sequence of the same length that are represented in the PBWT. Formally, given *w*-length input sequences $S=\{S_{1},\ldots ,S_{h}\}$ (sorted in M) and a pattern *P*[1.*w*], we define *P*[*i*.*j*], where 1≤i≤j≤w, to be an SMEM if it occurs in one of the input sequences of *S* and one of the following holds: (i) i=1 and j=w; (ii) i=1 and P[1..j+1] do not occur in *S*; (iii) j=w and P[i−1..w] do not occur in *S*; and (iv) P[i−1..j] and P[i..j+1] do not occur in *S*.

**Figure 1.**
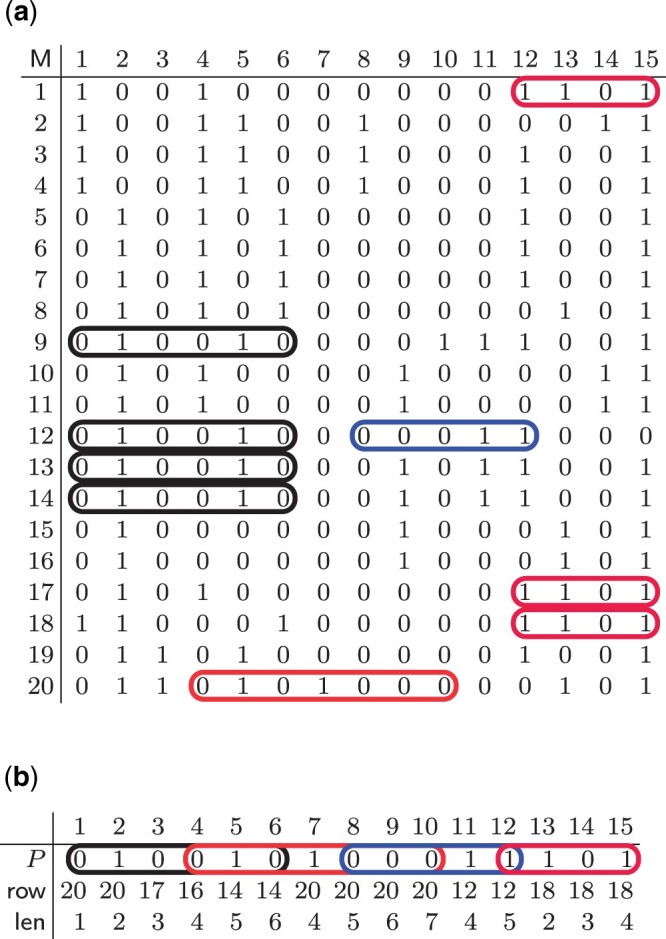
The input matrix M of 20 individuals of 15 bi-allelic sites (a), a query pattern *P* and its matching statistics with respect to M (b), SMEMs are circled in both the pattern and the input matrix M. (a) Input matrix M. (b) Pattern *P* with matching statistics.

SMEMs between a pattern *P* and a PBWT of a matrix M are illustrated in Fig. 1a and b: they are circled in both the pattern and the input matrix M.

We next define two problems related to finding the SMEMs. First, we define the problem of identifying the SMEMs in the pattern *P*.

Problem 1 (SMEM finding) Given a set $S=\{S_{1},\ldots ,S_{h}\}$ of *h* sequences of length *w* and a pattern *P*[1..*w*], compute the list *L* of pairs (p,ℓ) such that for all (p,ℓ)∈L, P[p..p+ℓ−1] are the SMEMs between *S* and *P*.

Then, we define the problem of locating all the occurrences of the SMEMs in the panel.

Problem 2 (SMEM locating) Given a set $S=\{S_{1},\ldots ,S_{h}\}$ of *h* sequences of length *w* and a pattern *P*[*1..w*], find the list *L* of triples (p,ℓ,O) such that for all (p,ℓ,O)∈L, P[p..p+ℓ−1] is an SMEM between *S* and *P* where *O* is the list of haplotypes where the SMEM occur.

Durbin’s Algorithm 5 (Durbin 2014) is able to solve Problem 2 in O(w)-time and O(n)-space, which corresponds to about 13*n* bytes. This memory consumption is the major downside of this algorithm and the motivation that led us to develop a run-length encoded PBWT that supports SMEMs finding and locating. For example, in Fig. 1a, we have 9 SMEMs computed by the pattern *P* in Fig. 1b.

## 3 Materials and methods

Our main contribution is a significant reduction in the memory used to store the PBWT via efficient sampling and storing the PA and DA arrays. In particular, we reduce the space of Durbin’s PBWT, which is O(n) space, to O(r) space. And while most of the PBWT operations which require O(1) time in Durbin’s PBWT—which explicitly stores the input matrix and the associated divergence and prefix arrays—take O(log r) time, this runtime is not observed in practice. We point to the experimental result for illustration of this fact in Section 4. Lastly, we refer the reader to Bonizzoni *et al.* (2022) for a more thorough evaluation of the data structures for the PBWT that support different time/space trade-offs for SMEM-finding.

### 3.1 Overview of μ-PBWT and data structures

Our solution to Problem 1, i.e. SMEM finding, in O(r) space requires three data structures: (i) a *mapping structure* to support the navigation of the RLPBWT; (ii) the *samples* of the prefix array (PA) in correspondence of the beginning and end of each run in the RLPBWT; and (3) the *thresholds* identifying the positions of the first minimum divergence array (DA) value in each run in the RLPBWT. We observe that the O(r) space is exactly the memory bound for all these data structures. First, we observe that the problem of finding SMEMs can be cast into the problem of computing matching statistics for *P* (Bannai *et al.* 2020), as described in the following. Let us first give the definition of matching statistics of a pattern with respect to a set *S* of sequences. Given a pattern *P*[1.*w*], the matching statistics of *P* with respect to the input set $S=S_{1},\ldots ,S_{h}$ of sequences are an array *A*[1.*w*] of (row,len) pairs such that, for each position *j*, with 1≤j≤w, A[j].row is the index of a sequence in *S* whose prefix ending in *j* has the longest suffix in common with *P*[1.*j*], and A[j].len is the length of that common suffix. More formally, we have $S_{A[j].\mathrm{row}}[j-A[j].\mathrm{len}+1..j]=P[j-A[i].\mathrm{len}+1..j]$ and for all $k\;S_{k}[j-A[j].\mathrm{len}..j]\neq P[j-A[i].\mathrm{len}..j]$.

SMEMs can be computed from the matching statistics for the PBWT as follows. We scan the matching statistics from right to left, and report a SMEM at the column j−A[j].len+1 (of the input matrix) of length A[j].len if either j=w, or A[j].len≥A[j+1].len. Informally, A[j].len≥A[j+1].len occurs when we cannot extend to the right the current longest common suffix (of length A[j].len) shared by *P* and any row in the input matrix. We show in Fig. 1b an example of matching statistics for the input matrix M representing the set *S* of sequences. Note that vector *A* is represented in two different lines, one for the row values and one for the len values.

Next, we show how to compute the matching statistics in O(r) space by storing the data structures mentioned before. Finally, we show how to solve Problem 2 in O(r) space of a small data structure that we refer to as Φ for the PBWT.

### 3.2 Finding SMEMs in μ-PBWT

As previously mentioned, our solution to finding SMEMs in O(r) space requires three data structures, which we now describe.

#### 3.2.1 Mapping structure

Given the position of a bit σ in the PBWT, say the *i*th row and *j*th column, our mapping data structure returns the positions in the next column of the PBWT of the bits immediately to the right in M. This is equivalent to forward stepping in the PBWT:

$$\mathrm{FL}[i][j]=\{\begin{array}{cc} u_{j}[i]+1 & \;\text{if}\;\sigma =0\\ v_{j}[i]+c[j]+1 & \;\text{if}\;\sigma =1 \end{array}$$

where (i) $u_{j}[i]$ is the number of zeros until *i* in $\mathrm{col}{\left(\mathrm{PBWT}\right)}_{j}$; (ii) $v_{j}[i]$ is the amount of ones until *i* in $\mathrm{col}{\left(\mathrm{PBWT}\right)}_{j}$; and (iii) *c*[*j*] is the total amount of zeros in $\mathrm{col}{\left(\mathrm{PBWT}\right)}_{j}$, as in Durbin’s paper.

This mapping allows us to step from one column to the next one (to the right) in the PBWT. Here, we remind the reader that due to the co-lexicographical ordering on the PBWT, it follows that FL mapping and forward stepping is the analogous counterparts of the LF mapping and the backward stepping in the BWT. Summarizing, for each column *j* in the RLPBWT, we store (i) the $r_{j}$ run head indices $p_{j}$, (ii) a single *r*-length data structure $uv_{j}$ for both $u_{j}$ and $v_{j}$, (iii) the integer *c*[*j*], and (iv) a Boolean value of *b* storing the symbol of the first run. Therefore, we demonstrate that mapping structure requires O(r) space.

In particular, the representation $uv_{j}$ for both $u_{j}$ and $v_{j}$ consists of an interleaved representation for each integer *i*, with 1≤i≤r of the value $v_{j}$ (or $u_{j}$, respectively), up to the start of run *i*, if the *i*th run consists of zeros (or ones, respectively). For example, given $\mathrm{col}{\left(\mathrm{PBWT}\right)}_{j}=00101111000000000000$ (with r=5), we store: (i) $p_{j}=[1,3,4,5,9]$, (ii) $uv_{j}=[0,2,1,3,5]$, (iii) c[j]=15, and (iv) $b_{j}=⊤$.

#### 3.2.2 PA samples and thresholds

Given the RLPBWT, we store the positions of the first minimum divergence array (DA) value for each run in each column of the RLPBWT. We refer to these as *thresholds*. More formally, let $\mathrm{col}{\left(\mathrm{PBWT}\right)}_{k}[i..j]$ be a maximal run in the *k*th column of the PBWT, we store the PA sampled at run boundaries, i.e. the values of $PA_{k}[i]$, $PA_{k}[j]$. We used bit-compressed integer vectors to store both PA samples and thresholds in O(r) space.

#### 3.2.3 Computing the matching statistics

Given our data structure, we show how to compute the matching statistics using an algorithm similar to the one used by Rossi *et al.* (2022), which computes the matching statistics in the BWT. In particular, we compute the matching statistics in a two-pass algorithm over the input pattern *P*. During the first scan, we process the pattern *P* from left to right, storing for each position the row component of the matching statistics. In the second scan, we process the pattern *P* from right to left, and, with the use of a random access data structure on the binary array M, we compute the len component of the matching statistics. We assume that we computed the matching statistics component up to position k−1 and are processing the *k*th column. We let *i* be the row of the PBWT that matches the longest suffix of P[1..k−1] that is the suffix of $S_{1}[1..k-1],\ldots ,S_{h}[1..k-1]$, and let *p* be the corresponding row in M, i.e. for all j∈[1..h], $\mathrm{lcs}(P[1..k-1],S_{PA_{k}[i]}[1..k-1])\geq \mathrm{lcs}(P[1..k-1],S_{PA_{k}[j]}[1..k-1])$ with $p=PA_{k}[i]$ where lcs(S,T) denotes the longest common suffix between two sequences *S* and *T*. Then, we distinguish two cases: *match in kth column*, i.e. when $\mathrm{col}{\left(\mathrm{PBWT}\right)}_{k}[i]=P[k]$ and *mismatch in kth column*, i.e. when $\mathrm{col}{\left(\mathrm{PBWT}\right)}_{k}[i]\neq P[k]$. If we have a match, then row i can be used to extend the suffix of P[1..k−1] to *P*[1.*k*]; hence, we can assign A[k].row=p, A[k].len=A[k−1].len+1, i=FL[i][k], and *p* does not change. Otherwise, if we have a mismatch, it means that for extending the suffix of P[1..k−1] to *P*[1.*k*], we need to move to a run before or after the one containing row *i* in $\mathrm{col}{\left(\mathrm{PBWT}\right)}_{k}$, as the value P[k]=col(PBWT)k[i]. Thus, let $\mathrm{col}{\left(\mathrm{PBWT}\right)}_{k}[s..e]$ be a maximal run containing position *i*, then the longest suffix of *P*[1.*k*] that is a suffix of $S_{1}[1..k],\ldots ,S_{h}[1..k]$ is either the one corresponding to the preceding end or following start of a run of value *P*[*k*] in $\mathrm{col}{\left(\mathrm{PBWT}\right)}_{k}$ with respect to position *i*, i.e. either $S_{PA_{k}[s-1]}[1..k]$ if s>1 or $S_{PA_{k}[e+1]}[1..k]$ if e<n. Since for each run we have stored the samples of PA at the beginning and at the end of each run, and we have the value of *p*, we can use the thresholds to decide which candidate to choose. Let *t* be the position of the threshold in the current run. Indeed the thresholds by definition report the positions of the first minimum divergence array (DA) value in each run. More precisely, if the position *t* is such that i<t it means that $\mathrm{lcs}(P[1..k],S_{PA_{k}[s-1]}[1..k])\geq \mathrm{lcs}(P[1..k],S_{PA_{k}[e+1]}[1..k])$ and we can assign $A[k].\mathrm{row}=p=PA_{k}[s-1]$ and i=FL[s−1][k]. Otherwise, $\mathrm{lcs}(P[1..k],S_{PA_{k}[s-1]}[1..k])\leq \mathrm{lcs}(P[1..k],S_{PA_{k}[e+1]}[1..k])$ hence we can assign $A[k].\mathrm{row}=p=PA_{k}[e+1]$ and i=FL[e+1][k].

Once we have collected all the occurrences of maximal matches between the pattern and the matrix, we can compute the lengths of those matches by scanning (using the reverse of the FL mapping) the pattern *P* from right to left and by comparing the characters in the pattern *P* and in the matrix in correspondence of row A[i].row.

Having that mapping structure, thresholds, and PA samples require O(r)-space, and matching statistics computation requires O(r)-space. An illustration of the computation of the matching statistics is shown in Supplementary Fig. S1a.

### 3.3 Locating SMEM in μ-PBWT

We note that although it is reasonably straightforward to report the number of occurrences of a given SMEM in *S*, it is more challenging to find the location of all the occurrences in *S*. To accomplish this, we store a small data structure that answers queries of the form: given a column index *k* and a prefix array value *j*, return the previous and the next prefix array value in that column, that is the row preceding and following *j* in co-lexicographic ordering up to column k−1. We observe that these two values correspond to rows that we need to consider for finding common suffixes with row *j*—and thus, the occurrence(s) of a SMEM in *S*. We refer to these as Φ-queries in the PBWT and the inverse of Φ, respectively, that will be denoted as $\Phi^{-1}$. More formally, given a value of $PA_{k}$ in position *i*, the Φ function returns the preceding value of $PA_{k}$ in position i−1, and the $\Phi_{k}^{-1}$ function returns the value of $PA_{k}$ in position i+1. Our contribution is the implementation in PBWT of the Φ function (Kärkkäinen *et al.* 2009) and its inverse by storing the smallest information to compute them. As an example of Φ, assuming $PA_{6}=[15,16,18,1,5,6,7,8,10,11,17,9,12,13,14,19,20,2,3,4]$ and the PA value 19, we have that $\Phi_{6}(19)=14$ and $\Phi_{6}^{-1}(19)=20$.

Since our goal is the computation of the Φ function by keeping only PA samples, we show how performing the FL mapping allows to compute the values of the Φ that are of interest. We observe that if we perform the FL mapping in a column of the PBWT of two consecutive equal symbols (0 or 1), the resulting positions of the haplotypes in the PBWT are consecutive in the next column after the mapping and their relative order is preserved.

Formally, for all 1≤j<w and for all 1≤i<h, if $\mathrm{col}{\left(\mathrm{PBWT}\right)}_{j}[i]=\mathrm{col}{\left(\mathrm{PBWT}\right)}_{j}[i-1]$ then FL[i][j]=FL[i−1][j]+1=l, for some row *l*, and therefore, $PA_{j}[i]=PA_{j+1}[l]$ and $PA_{j}[i-1]=PA_{j+1}[l-1]$. This implies we can compute the value of $\Phi (PA_{j}[i])$—i.e. compute the value of $PA_{j}[i-1]$—by performing an FL mapping as long as the corresponding PBWT values are the same. By the above observation, we only need to store the PA sample at the beginning and at the end of each PBWT run since this position will correspond to the first mismatch in the PBWT occurring by performing a FL mapping. More precisely, assuming that *k* is the column to the right of *j* and $i^{\prime }$ is the row corresponding to the PBWT values mismatch reached by FL mapping, i.e. col(PBWT)k[i′]=col(PBWT)k[i′−1], then, we have that $PA_{k}[i^{\prime }]=PA_{j}[i]$ and $PA_{k}[i^{\prime }-1]$ is sampled since it is at the end of a run. Therefore, by the above observation, we can retrieve the value of $PA_{j}[i-1]=PA_{k}[i^{\prime }-1]$.

An example of iterative FL mapping to perform Φ queries is depicted in Supplementary Fig. S1b, with brown rounded boxes. Suppose that in column 6 we have the prefix array value 19 (at i=16) and we want to compute $\Phi_{6}(19)=14$. As in Supplementary Fig. S1a, we have that $\mathrm{col}{\left(\mathrm{PBWT}\right)}_{6}[16]=\mathrm{col}{\left(\mathrm{PBWT}\right)}_{6}[15]$ but we do not have $PA_{6}[15]$ in memory. Performing FL mapping starting from row 15th and from row 16th in Column 6, we reach Row 10 and Row 11, respectively, in Column 7, having that $\mathrm{col}{\left(\mathrm{PBWT}\right)}_{7}[10]=\mathrm{col}{\left(\mathrm{PBWT}\right)}_{7}[11]$. The same happens when moving from Column 7 to Column 8 ($\mathrm{col}{\left(\mathrm{PBWT}\right)}_{8}[10]=\mathrm{col}{\left(\mathrm{PBWT}\right)}_{8}[11]$). At this point, we do not yet know $PA_{7}[10]$ and $PA_{8}[10]$. Instead, reaching Column 9, we have $\mathrm{col}{\left(\mathrm{PBWT}\right)}_{9}[10]\neq \mathrm{col}{\left(\mathrm{PBWT}\right)}_{9}[11]$. In detail, in Row 10, we have the tail of a run of bits σ=1 and in Row 11 the head of a run of bits σ=0 and, as explained above, we store PA values at round boundaries, so we can get the value 14 from PA samples, being the prefix array value that preceded the value 19 also in Column 6 (as well as in Columns 7 and 8).

We observe that we can use at this point the DA samples together with the information of the current row of an SMEM and the next/previous row retrieved by Φ function, to directly check if also the latter shares the same SMEM. If we have an SMEM in column *k*, we can follow the *A[k]*.*pos* to column k+1 and we can analyze the rows adjacent to it in the co-lexicographic order, which is up to column *k*th, to compute which other rows share the same SMEM, having that all the rows that share the same SMEM in *k* will be consecutive in $PA_{k+1}$. Therefore, if we store the DA sample at the beginning of each PBWT run, while computing the Φ function for $PA_{j}[i]$, we can recover the value of $DA_{j}[i]$ as $DA_{k}[i^{\prime }]-(k-j)$, that is removing from the sampled value $DA_{k}[i^{\prime }]$ the distance traveled by the repeated application of the FL mapping.

For example, consider the SMEM in Fig. 1b identified by A[6].pos=14 and A[6].len=6. Assume $PA_{7}=[15,16,1,10,11,17,9,12,13,14,19,20,2,3,4,18,5,6,7,8]$ and $DA_{7}=[0,6,2,4,6,6,1,6,6,6,3,6,2,6,6,0,2,6,6,6]$ (full prefix array set available in Supplementary Fig. S1). Since $\Phi_{7}(14)=13$ and $DA_{7}[10]=6$ (having $PA_{7}[10]=14$) then it follows that we know 13th row shares the same SMEM, similar to Row 12 and Row 9. Then, we iterate until $\Phi_{7}(9)=17$, having that $DA_{7}[7]=1$. Using $\Phi_{7}^{-1}$, we reach 19th row ($\Phi_{7}^{-1}(14)=19$) but $DA_{7}[11]=3$, which is less than A[6].len=6—so we do not have any other row that shares this SMEM.

More details on the computation of the Φ function are given in the Supplementary Material.

We observe that the space-bound O(r) follows from the fact that the information needed to compute Φ function is given by a sparse bitvector representation of the PA and DA samples.

## 4 Results

We demonstrate the performance of μ-PBWT by comparing μ-PBWT with: Durbin’s Algorithm 5 (implemented as matchIndexed in the official source code) and Syllable-PBWT (Wang *et al.* 2023). More precisely, for Durbin’s Algorithm 5, we will evaluate (i) the memory usage peak and (ii) the time required for SMEM finding. For Syllable-PBWT, we will evaluate (i) the memory usage peak for index construction and (ii) the size of the index. We could not compare μ-PBWT performance in SMEMs-finding with Syllable-PBWT since Syllable-PBWT implementation allows to compute only L-long matches that are matches length at least L sites. Therefore, L-long matches are a superset of SMEMs. Finally, we report some statistical results on μ-PBWT. We note that a compact file format for storing haplotype sequences based on the PBWT was proposed in Li (2016); however, the proposed format does not support SMEM queries for external haplotypes.

### 4.1 Implementation details

μ-PBWT is implemented in C++17 using standard library data structures and relying on the Succinct Data Structure Library (sdsl) (Gog *et al.* 2014) for succinct data structures implementations such as int_vectors and sd_vectors with rank and select support. VCF and BCF files input files are supported using the htslib library (Bonfield *et al.* 2021).

### 4.2 Experimental setup

We demonstrate the performance of μ-PBWT on real-world datasets. We report the time and memory used for construction and SMEM-locate queries.

We ran experiments on a machine with an Intel Xeon CPU E5-2640 v4 (2.40 GHz), 756 GB RAM, and 768 GB of swap, running Ubuntu 20.04.4 LTS (64 bit, kernel 5.4.0). The compiler was g++ version 9.4.0 with -O3 option. The running time and the maximum resident set size were computed by /usr/bin/time.

### 4.3 Datasets

We first tested μ-PBWT on all chromosome panels from the 1000 Genome Project. The VCF files were downloaded (publicly available at https://ftp.1000genomes.ebi.ac.uk/vol1/ftp/release/20130502/) and converted to contain only bi-allelic sites via bcftools view -m2 -M2 -v snps (Danecek *et al.* 2021). The resulting chromosome panels have 5008 haplotypes and a number of bi-allelic sites ranging from ∼1 million to ∼6 million. Statistics of the 1000 Genome project panels are in Supplementary Table S3. Experimentally, we observed these panels are sparse, having indeed fewer ’1s compared to ’0s. The sparsity of data is confirmed by the average number 11 of runs per column in the run-length encoded PBWT.

We used UK Biobank high-coverage WGS data on chromosome 20 (Bycroft *et al.* 2018). More precisely, we consider data available on the UK Biobank research analysis platform (Halldorsson *et al.* 2022) recently processed and phased by the SHAPEIT5 authors (Hofmeister *et al.* 2023), for a total of 300 238 haplotypes and 13 780 193 bi-allelic SNPs and indels on chromosome 20. For the UK Biobank WGS dataset, we applied our method independently to 13 regions of at least 4 megabases and 4 centimorgans on chromosome 20. Additional results on simulated data are reported in the Supplementary Material.

### 4.4 Results on 1000 genomes project data

**Figure 2.**
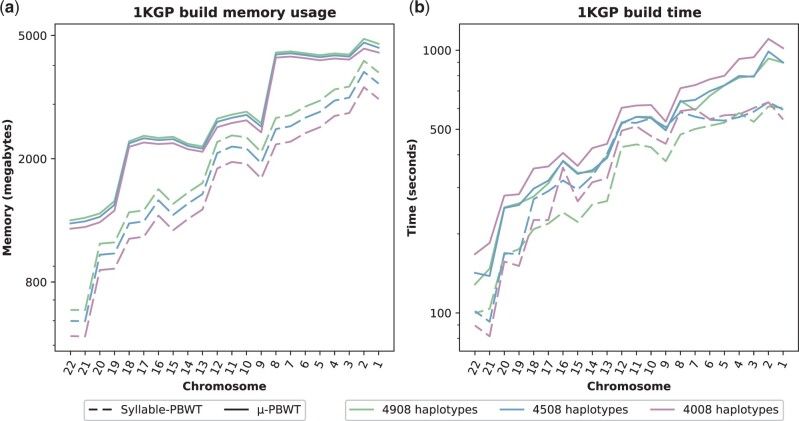
Comparison of the construction of the indexes on 1000 genome Project data with 4908, 4508, and 4008 haplotypes. In (a), we have maximum memory usage and, in (b), we have time results. PBWT is excluded as most indices are calculated at query time

In Fig. 2a, we report the memory peak during construction of μ-PBWT and Syllable-PBWT while in Fig. 2b the time. Comparison with the construction of the PBWT has been excluded as most indices are calculated at query time. We note that Syllable-PBWT performs slightly better than μ-PBWT, taking about half of the memory and computation time. We note that our indices, as in Supplementary Table S3, require only twice the memory compared to the input and they require 25% less memory than the indices of Syllable-PBWT.

**Figure 3.**
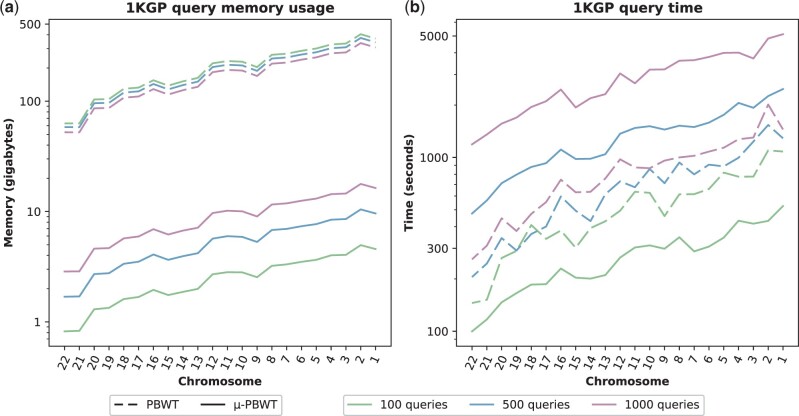
Comparison on the 1000 genome Project data for finding SMEMs for 100, 500, and 1000 queries. In (a), we have maximum memory usage and, in (b), we have time results. Syllable-PBWT is excluded as it does not compute SMEMs

To test the performance of computing SMEMs, 100, 500, and 1000 haplotypes, respectively, were extracted from the input panels (reduced to 4908, 4508, and 4008 haplotypes), to use them as queries. In Fig. 3a, we report the memory peak during SMEMs finding of μ-PBWT and PBWT while in Fig. 3b, the time. Comparison with the construction of Syllable-PBWT has been excluded as it computes L-long matches instead of SMEMs. Regarding μ-PBWT, memory usage increases as the number of queries increases, since the entire set of queries is kept in memory. Our SMEMs-finding algorithm is up to six times slower than the one proposed by Durbin although as the number of queries decreases, it turns out to require twice the time. Observe that for a fair comparison with Durbin, since Durbin’s PBWT builds most of the indices at query time, we had to measure the time of μ-PBWT for building and querying the index. Increased time of μ-PBWT however comes with a significantly lower memory usage, as Durbin’s PBWT memory peak is up to 80 times the one of μ-PBWT.

Then we analyzed the stratification of the memory usage of μ-PBWT for the mapping structure, PA/DA samples, thresholds, and Φ data structure. Note that the Φ data structure is the component that requires slightly more amount of memory, about 40% of the total since it stores two sparse bitvectors panels and three bit-compressed int-vectors that scale with the total number of runs of the PBWT. Then we have PA/DA samples requiring about 30% of memory, mapping structure requiring about 20%, and finally thresholds requiring about 10% of memory, as expected, being for each column a single bit-compressed intvector of length equal to the number of runs.

Additional results on 1000 Genome Project data, including BGT (Li 2016) comparison and multithreads μ-PBWT, are reported in the Supplementary Material.

### 4.5 Results on UK Biobank data

We also applied our method to the UK Biobank high-coverage WGS data on chromosome 20. In this setting, our method is able to build an index for the full chromosome 20 in 11.06 GB of space that represents an almost three times decrease compared to the original gzipped BCF files (stored in a 29.6 GB file), highlighting the potential of our method for compressed genomics on next-generation datasets. Full results are available in Supplementary Table S2. Syllable-PBWT takes as input only raw (not gzipped) VCF files, preventing a comparison with UK Biobank data due to BCF decompression time and the disk space required for storing the VCF file required for using Syllable-PBWT (even if the panel was divided into multiple regions).

All the indices generated by μ-PBWT are loaded <30 s on a commodity laptop (AMD Ryzen7 3700U and 16 GB RAM), drastically reducing the hardware requirements for data sharing and analysis of WGS data.

## 5 Conclusions

In this article, we present μ-PBWT, introducing a lightweight index for the PBWT data structure. It leverages the run-length encoding paradigm to significantly reduce the space requirements for solving two major problems: the SMEMs finding (i.e. computing maximal matches) and SMEMs location (i.e. finding occurrences). The main idea behind our method is that μ-PBWT stores only the information needed to navigate the PBWT by leveraging the runs of haplotypes. Compared to the investigation of the use of the BWT for large genomics data, the PBWT has been comparatively overlooked by the data structures community, even though the increased demand of tools for managing large phased datasets, such as the UK Biobank WGS data, for which the PBWT has been originally proposed, making the urgent need of space efficient solutions to store and use these data. Results on UK Biobank WGS data suggest that μ-PBWT can scale on whole genome genotype data and it can be used for applications on very large and repetitive datasets that require SMEMs finding such as in phasing and imputation (Rubinacci *et al.* 2020).

## Supplementary Material

btad552_Supplementary_Data
