# Supplementary material for: μ- PBWT: a lightweight r-indexing of the PBWT for storing and querying UK Biobank data
Source: Bioinformatics. 2023 Sep 9;39(9):btad552. doi: 10.1093/bioinformatics/btad552 (PMC10502237; doi:10.1093/bioinformatics/btad552)
Supplement: btad552_Supplementary_Data [file btad552_supplementary_data.pdf]

Supplementary Material

Additional examples

Figure S1 shows additional examples related to the input matrix in Figure 1.

(a) PBWT.

	1	2	3	4	5	6	7	8	9	10	11	12	13	14	15
1	1	1	0	1	1	0	0	0	1	0	0	1	1	1	1
2	1	1	0	1	1	0	0	0	1	0	0	1	1	1	1
3	1	1	0	1	1	1	0	0	0	1	1	1	0	1	1
4	1	1	0	1	1	0	0	0	1	0	0	1	1	0	1
5	0	1	0	1	0	1	0	0	1	0	0	1	1	0	1
6	0	1	0	1	0	1	0	0	0	0	0	1	0	0	1
7	0	1	0	1	0	1	0	0	0	0	0	1	0	0	0
8	0	1	0	1	1	1	0	0	0	0	0	0	1	0	1
9	0	1	0	0	1	0	0	0	1	0	0	0	1	0	1
10	0	1	0	1	0	0	0	0	1	0	0	0	0	0	1
11	0	1	0	1	1	0	0	0	0	0	0	1	1	0	1
12	0	1	0	0	1	0	1	1	0	0	0	1	0	0	1
13	0	1	0	0	1	0	0	1	0	0	0	0	0	0	1
14	0	1	0	0	0	0	0	1	0	0	0	0	0	0	1
15	0	1	0	0	0	0	0	0	0	0	0	0	0	0	1
16	0	0	0	0	0	0	0	0	0	0	0	0	0	0	1
17	0	0	0	1	0	0	0	0	0	0	0	1	0	0	1
18	1	0	1	0	0	0	0	0	0	0	1	1	0	0	1
19	0	0	1	0	0	0	0	0	0	0	1	1	0	0	1
20	0	1	0	0	0	0	0	0	0	0	1	1	0	0	1

(b) Prefix array.

	1	2	3	4	5	6	7	8	9	10	11	12	13	14	15
1	1	5	1	1	9	15	15	15	15	1	1	1	8	2	19
2	2	6	2	2	12	16	16	16	16	17	17	17	20	10	5
3	3	7	3	3	13	18	1	1	1	9	12	19	2	11	6
4	4	8	4	4	14	1	10	10	10	12	19	18	15	19	7
5	5	9	5	5	15	5	11	11	11	19	18	5	16	5	3
6	6	10	6	6	16	6	17	17	17	18	5	6	10	6	4
7	7	11	7	7	18	7	9	9	9	5	6	7	11	7	12
8	8	12	8	8	19	8	12	12	12	6	7	8	1	3	13
9	9	13	9	9	20	10	13	13	13	7	8	20	17	4	14
10	10	14	10	10	1	11	14	14	14	8	20	2	19	12	9
11	11	15	11	11	2	17	19	19	19	20	2	3	18	13	8
12	12	16	12	12	3	9	20	2	18	2	3	4	5	14	20
13	13	17	13	13	4	12	2	3	5	3	4	15	6	9	15
14	14	19	14	14	5	13	3	4	6	4	15	16	7	8	16
15	15	20	15	15	6	14	4	18	7	15	16	10	3	20	1
16	16	1	16	16	7	19	18	5	8	16	10	11	4	15	17
17	17	2	17	17	8	20	5	6	20	10	11	12	12	16	18
18	18	3	19	18	10	2	6	7	2	11	13	13	13	1	2
19	19	4	20	19	11	3	7	8	3	13	14	14	14	17	10
20	20	18	18	20	17	4	8	20	4	14	9	9	9	18	11

Fig. S1. The PBWT (a) of M (see Figure 1) and the corresponding prefix array (PA) (b). We illustrate the SMEM-finding procedure via the computation of matching statistics. We start from an arbitrary row. In this case, we choose the 20th, where $\mathsf{col}(\mathsf{PBWT})_1[20] = 0$. Since we have that $P[1] = 0$, we proceed to the next column and store $\mathsf{A}[1].\mathsf{row} = 20$ and $\mathsf{A}[1].\mathsf{len} = 1$. To advance by column, we compute the mapping function of row 20 from the first column to the second. Observe that the mapping function is used to compute index k of $\mathsf{col}(\mathsf{PBWT})_{i+1}$ that contains $A[i].\mathsf{row}$. Hence, the result is that we are mapping to $\mathsf{col}(\mathsf{PBWT})_2[15]$. At the second column, we have $P[2] = \mathsf{col}(\mathsf{PBWT})_2[15]$, so we can proceed to the next column, storing $\mathsf{A}[2].\mathsf{row} = 20$ and $\mathsf{A}[2].\mathsf{len} = 2$. Following the mapping of the row 20, we move onto $\mathsf{col}(\mathsf{PBWT})_3[19]$. We have a mismatch at column 3 since $P[3] \neq \mathsf{col}(\mathsf{PBWT})_3[19]$. At this point, we can move to either the last character of the previous run, $\mathsf{col}(\mathsf{PBWT})_3[17]$, or the first character of the next run, $\mathsf{col}(\mathsf{PBWT})_3[20]$, having $\mathsf{PA}_3[17] = 17$ and $\mathsf{PA}_3[20] = 18$. If we look at the input matrix M, we have that, up to column 3 excluded, row 17 has a common suffix to row 20 longer than row 18. So, the best option to maximize the length of the current match is to move to row 17, storing $\mathsf{A}[3].\mathsf{row} = 17$ and $\mathsf{A}[3].\mathsf{len} = 3$. Now we use row 17 to compute the mapping function from column 3 to column 4. We proceed in this way until we complete the computation of A. Finally, with a sweep from left to right over A, we can compute all the SMEMs looking at the indices where $\mathsf{A}[i].\mathsf{len} \geq \mathsf{A}[i+1].\mathsf{len}$, as shown through the colored rounded boxes covering P in Figure 1.

Φ data structure

Figure S2 represents how the Φ data structure is computed by using the FL mapping. To avoid performing $\mathcal{O}(k - j - 1)$ FL steps, it is possible to store a successor data structure maintaining the columns where the haplotypes appear as PA sample at the beginning of a run and storing the corresponding sample at the end of the previous run and the DA sample as satellite information.

Results on UK Biobank

In Table S2 we summarize the results of UK Biobank high-coverage whole genome sequencing data on chromosome 20.

In addition, we analyzed UK Biobank SNP array data. We used UK Biobank SNP array data across all autosomes (any chromosome that is not a sex chromosome) and high-coverage whole genome sequencing data on chromosome 20 [26]. For the SNP array data, we applied the standard QC recommended by the original authors [26], and phased the data using SHAPEIT4 [28] resulting in 976,754 haplotypes and a total of 670,741 SNPs. Due to the low sparsity of these panels, this produced μ-PBWT with a high number of runs for each column, for example about 13462 for the chromosome 20 panel. We run in parallel μ-PBWT on all the 22 chromosomes, building our index in less than 2 hours. In Table S1 we summarize the results on UK Biobank SNP array panels.

Results on 1000 Genomes Project data and comparison with BGT

Here we graphically show additional results on 1000 Genome Project data, including BGT [22] indexing results and multithread μ-PBWT. We recall that BGT is not designed to perform matching queries but puts more emphasis on fast random access. In Table S3 we show some quantitative results on the indices comparison between μ-PBWT and Syllable-PBWT, in Figure S3 we show the memory usage of every component of μ-PBWT, using 4908 haplotypes, in S4 we add BGT to the comparison of results for constructing the indices, in S5 we add μ-PBWT SMEMs finding with 12 threads (achieving even more similar performance to Durbin's PBWT) and in S6 we show indices and BCF/BGT file sizes.

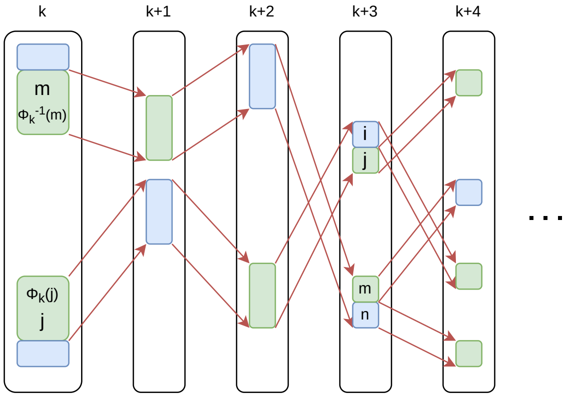

Fig. S2. Assume that with green blocks we denote bits of value 0 and with blue blocks bits of value 1. Suppose we want to compute $\Phi_k(j)$ i.e. the value placed above j in column k. Following these two equal bits in column $k+1$, using the FL mapping, we reach two positions that have two bits of the same value. The same happens when going from the $(k+1)$-th column to that $(k+2)$-th. As shown in the figure, another FL mapping iteration reaches two different bits in column $k+3$. In this column $\Phi_k(j)$ is at run boundary and we can extract its value i from PA samples, understanding that $\Phi_k(j)$ corresponds to the value i also at columns k, $k+1$ and $k+2$. In the symmetric case, $\Phi_k^{-1}(m)$ is computed as the value n, as illustrated in the figure.

Table S1. μ-PBWT results on UK Biobank SNP array. Columns from left to right report the chromosome number, the number of sites, the size of the input (in BCF), and the size of μ-PBWT indices. Each panel has 976754 haplotypes. The last two columns are measured in GB.

Chr	Sites	BCF	μ-PBWT
1	54432	4.2	13
2	53433	4.2	13
3	44935	3.6	11
4	41678	3.3	10
5	40020	3.2	9.6
6	46515	3.9	9.4
7	36682	3.0	8.9
8	34141	2.7	8.2
9	29581	2.4	7.7
10	33086	2.7	8.2
11	33827	2.7	7.8
12	32011	2.6	8
13	22344	1.9	6.3
14	21708	1.8	5.8
15	21286	1.8	6.1
16	24314	2.0	6.4
17	22856	1.9	6.2
18	19432	1.6	5.8
19	19845	1.6	5.3
20	17515	1.5	5.2
21	9940	0.8	3.4
22	11160	0.9	3.6

Results on msprime simulated panels

We simulated a 10 megabase region of European samples simulated with msprime [29] with an increasing number of haplotypes up to 2 million (10k, 100k, and 1000k individuals, namely panels 1,2 and 3). We also sub sampled the panel with 1000k individuals to obtain two additional panels, namely panels 4 and 5, with the same amount of sites but with 100k and 250k individuals. In Table S4 we have collected the quantitative data of these panels. The BCF files were pre-processed to contain only bi-allelic sites. Only the two smaller datasets (panel 1 and panel 2) allowed experimentation with Syllable-PBWT, which takes as input only raw (not gzipped) VCF files. Syllable-PBWT produced indices requiring up to 25 times more space, 16 times more memory and 16 times more time compared to μ-PBWT.

The results are displayed in Figure S8. As baseline, we also plot Durbin's Algorithm 5 estimations on memory usage. On the largest panel, μ-PBWT reduces the memory consumption by about 25000 times compared to the original PBWT implementation. The average number of runs in each column confirms the high sparsity of these simulated panels, achieving greater effectiveness in the use of run-length encoding and data structures that scale linearly (both in space and time) on the number of runs. In Figure S7 we report the stratification of the memory usage of the various components that make up μ-PBWT.

Table S2. UK Biobank high-coverage whole genome sequencing data on chromosome 20 information. Columns from left to right report the region, the number of samples, the number of sites, the size of the input (in BCF), the size of μ-PBWT indices, building time, and memory peak usage for construction. In the last row we report the total results. Regarding total build time consider that we have built μ-PBWT for every chromosome 20 region in parallel. The last four columns are measured in GB except for the second last which is measured in hh:mm.

Region	Samples	Sites	Size BCF	μ-PBWT	Time	Memory peak
chr20:60061-4060065	150119	865267	1.9	0.88	06:25	2.27
chr20:4060066-8060066	150119	880899	2	0.85	06:28	2.22
chr20:8060067-12515479	150119	961591	2.1	0.77	07:04	2.05
chr20:12515480-16768988	150119	917468	2	0.73	06:47	1.97
chr20:16768989-21050967	150119	931010	2	0.71	06:53	1.92
chr20:21050968-31549151	150119	1919134	4.2	1.20	13:54	3.06
chr20:31549152-38282825	150119	1436549	2.8	0.99	10:25	2.63
chr20:38282826-43181963	150119	1056144	2.2	0.76	07:42	2.06
chr20:43181964-47619489	150119	955970	2	0.79	06:56	2.09
chr20:47619490-51789198	150119	923178	2	0.80	06:44	2.12
chr20:51789199-55789212	150119	911452	2	0.81	06:45	2.13
chr20:55789213-59874964	150119	925442	2	0.84	06:49	2.20
chr20:59874965-64334101	150119	1096089	2.4	0.93	08:00	2.42
**Total**	**150119**	**13780193**	**29.6**	**11.06**	**-**	**29.15**

Table S3. 1000 Genome Project panels information. Columns from left to right report the chromosome number, the number of sites, the average number of runs for each column, the size of the input (in BCF), the size of μ-PBWT indices, and Syllable-PBWT indices. The last two columns are measured in GB. Each panel has 4908 haplotypes.

Chr	Sites	Runs	BCF	μ-PBWT	Syllable-PBWT
1	6196151	11	0.14	0.29	0.33
2	6786300	10	0.14	0.30	0.33
3	5584397	10	0.22	0.41	0.54
4	5480936	10	0.23	0.45	0.54
5	5037955	9	0.28	0.51	0.67
6	4800101	10	0.28	0.55	0.69
7	4517734	10	0.32	0.63	0.80
8	4417368	10	0.29	0.57	0.72
9	3414848	11	0.32	0.58	0.78
10	3823786	10	0.35	0.60	0.84
11	3877543	10	0.47	0.82	1.14
12	3698099	10	0.49	0.84	1.19
13	2727881	10	0.50	0.87	1.18
14	2539149	11	0.43	0.81	1.05
15	2320474	12	0.56	0.97	1.36
16	2596072	12	0.58	1.03	1.39
17	2227080	12	0.64	1.06	1.48
18	2171378	11	0.63	1.08	1.55
19	1751878	13	0.71	1.19	1.69
20	1739315	11	0.71	1.20	1.72
21	1054447	14	0.84	1.47	2.09
22	1055454	14	0.78	1.44	1.91

Table S4. μ-PBWT results on msprime simulated data. Columns from left to right report an ID, the number of sites, the number of samples, the average number of runs for each column, the size of the input (in BCF), the size of μ-PBWT indices and the size of Syllable-PBWT indices. The last three columns are measured in GB. We were not able to run all the experiments with the Syllable-PBWT due to disk limits, as the input format of Syllabe-PBWT is only uncompressed VCF files.

Panel	Samples	Sites	Runs	BCF	μ-PBWT	Syllable-PBWT
1	10000	209531	9	0.04	0.06	0.25
2	100000	743171	13	0.55	0.34	8.70
3	100000	2271035	4	1.2	0.49	-
4	250000	2271035	8	2.6	0.78	-
5	1000000	2271035	18	9.8	2.02	-

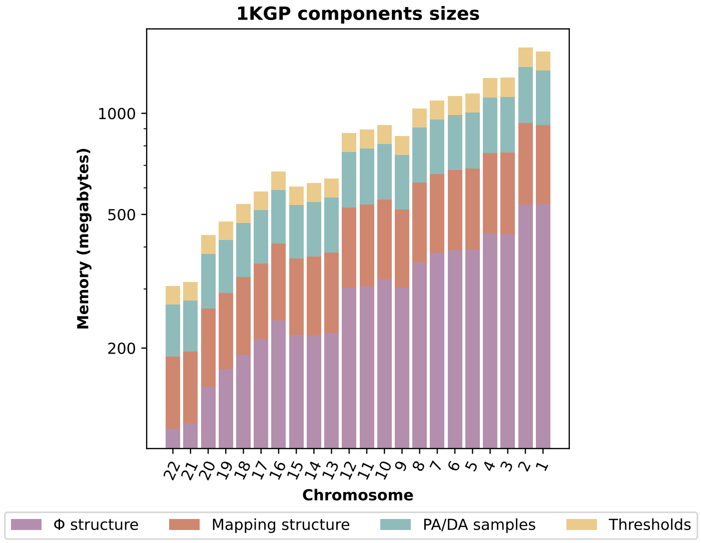

Fig. S3. Comparison of the results regarding memory usage of the main components of μ-PBWT on the 1000 Genome Project data. All the panels have 4908 haplotypes.

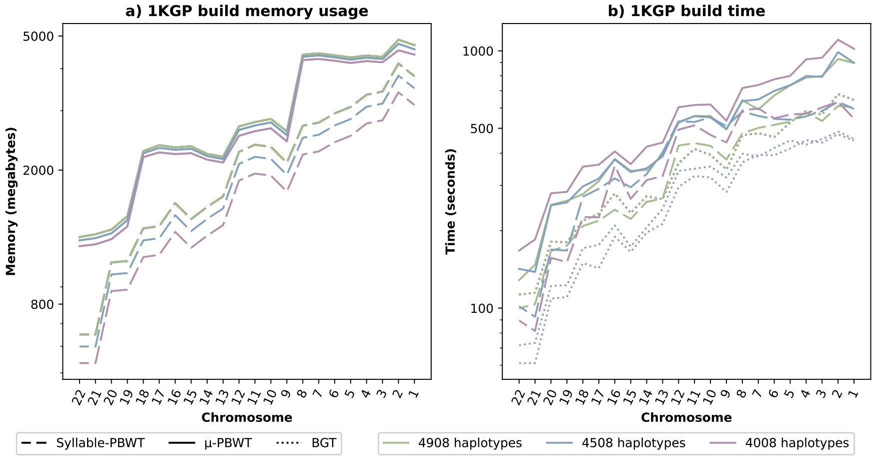

Fig. S4. Comparison of the results for constructing the index on 1000 Genome Project data with 4908, 4508, and 4008 haplotypes. In a) we have maximum memory usage and in b) we have time results. PBWT is excluded as most indices are calculated at query time.

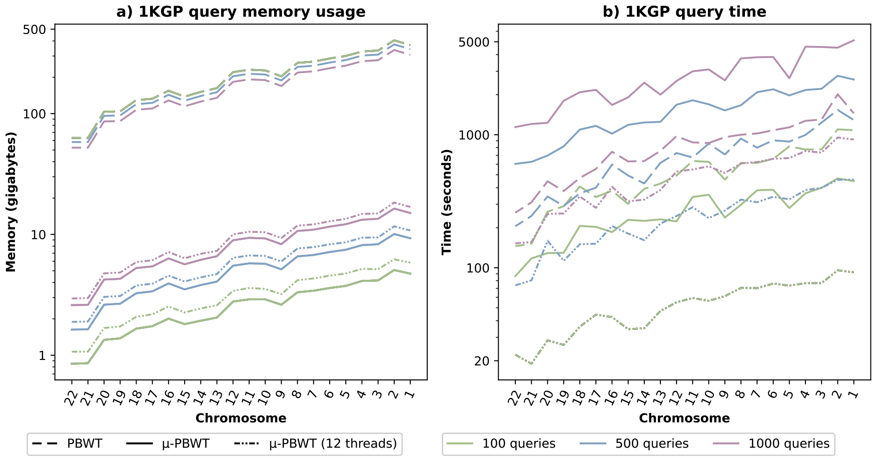

Fig. S5. Comparison of the results regarding SMEMs finding on 1000 Genome Project data with 100, 500, and 1000 queries. In a) we have maximum memory usage and in b) we have time results. Syllable-PBWT is excluded as it does not compute SMEMs.

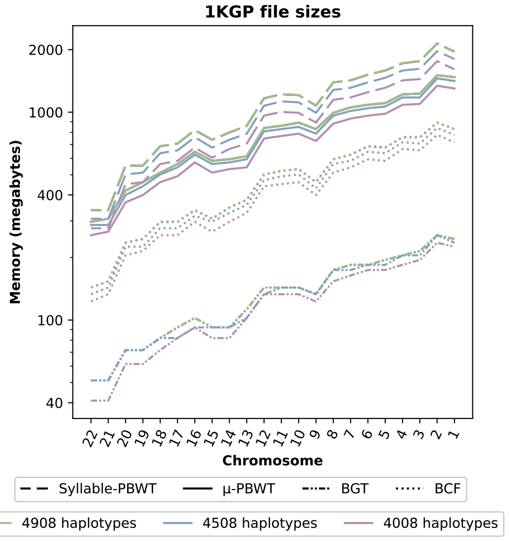

Fig. S6. Comparison of the results on 1000 genome Project data regarding BCFs, μ-PBWT indices, Syllable-PBWT indices, and compact files produced by BGT.

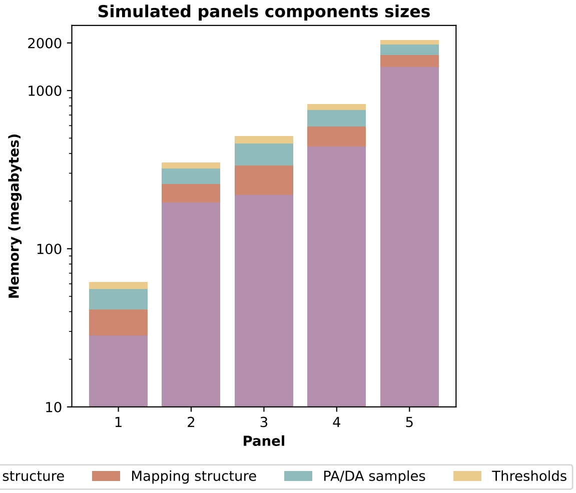

Fig. S7. Comparison of the results regarding memory usage of the main components of μ-PBWT on msprime simulated data.

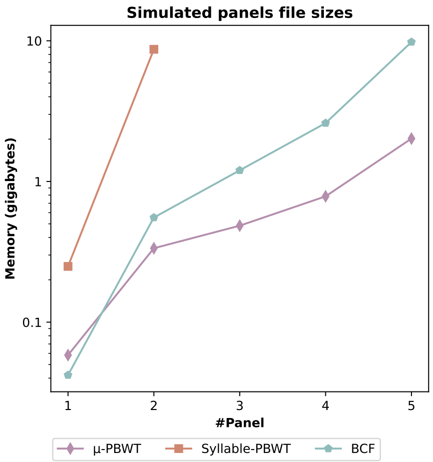

Fig. S8. Comparison on msprime simulated data regarding BCFs, μ-PBWT indices and Syllable-PBWT indices.
